# Phosphorylated AKT1 is associated with poor prognosis in esophageal squamous cell carcinoma

**DOI:** 10.1186/s13046-015-0212-z

**Published:** 2015-09-04

**Authors:** Zhengfei Zhu, Weiwei Yu, Xiaolong Fu, Menghong Sun, Qiao Wei, Dali Li, Haiquan Chen, Jiaqing Xiang, Hecheng Li, Yawei Zhang, Weixin Zhao, Kuaile Zhao

**Affiliations:** Department of Radiation Oncology, Fudan University Shanghai Cancer Center, 270 Dong An Road, Shanghai, 200032 China; Department of Radiation Oncology, Six Hospital of Jiao Tong University, Shanghai, China; Department of Pathology, Fudan University Shanghai Cancer Center, Shanghai, China; Department of Thoracic Surgery, Fudan University Shanghai Cancer Center, Shanghai, China; Department of Oncology, Shanghai Medical College, Fudan University, 270 Dong An Road, Shanghai, 200032 China

**Keywords:** Esophageal carcinoma, Esophageal squamous cell carcinoma, Epidermal growth factor receptor, p-AKT1, Prognostic factor, Prognosis, Immunohistochemistry

## Abstract

**Background:**

The epidermal growth factor receptor (EGFR) signaling pathway is important in regulating biological behaviors in many malignancies. We explored whether expression and activation of EGFR and several components on its downstream pathways have prognostic significance in patients with esophageal squamous cell carcinoma (ESCC).

**Methods:**

Expression of EGFR, phosphorylated (p)-EGFR, AKT1, p-AKT1, AKT2, p-AKT2, ERK1, ERK2, p-ERK1/2, STAT3, and p-STAT3 was assessed by immunohistochemical analysis of tissue microarrays for 275 ESCC patients who had undergone complete three-field lymphadenectomy. Spearman rank correlation tests were used to determine the relationships among protein expression, and Cox regression analyses were performed to determine the prognostic factors on overall survival (OS).

**Results:**

p-EGFR expression was correlated statistically with all of the other phosphorylated markers. Gender, N stage, and p-AKT1 expression were found to be independent prognostic factors for OS. Increased expression of p-AKT1 was associated with decreased patient survival. EGFR and p-EGFR expression was not significantly associated with patient survival.

**Conclusion:**

Activation of AKT1 was associated with poor prognosis in ESCC.

## Background

Esophageal cancer (EC) is a common malignancy worldwide, with Asia being one of the high-prevalence areas [1]. Although the incidence rates for esophageal adenocarcinoma have been increasing in several Western countries, esophageal squamous cell carcinoma (ESCC) is the most common histological type in Eastern countries, such as China, where it accounts for more than 90 % of EC cases [2]. Despite improvements in surgical techniques, perioperative management, and surgery combined with chemotherapy and/or radiotherapy, the prognosis for ESCC remains poor, particularly in advanced stages [3]. Therefore, the development of new therapy modalities, particularly targeted therapies based on knowledge of the biology and genetics of the disease, may offer the potential for improving treatment response and quality of life for ESCC patients.

In the past decade, great interest has been directed toward the use of agents targeting cell surface receptors that are responsible for the development and progression of various cancers. Epidermal growth factor receptor (EGFR) is one of the most commonly altered receptors in human malignancies. This receptor is mainly involved in regulating cellular processes including cell apoptosis, proliferation, angiogenesis, migration, and adhesion through activation of PI3K-Akt, STAT3, and Ras-Raf-MAPK signaling pathways [4]. In a variety of human cancers, increased expression of EGFR has been identified and shown to be associated with advanced disease, development of metastases, and poor clinical prognosis in a subset of these cancers [5]. However, study results on the prognostic effect of EGFR in ESCC remain conflicting [6–10]. To investigate the role of EGFR-related pathway activities in ESCC progression, we used tissue microarray (TMA) technology and immunohistochemical (IHC) analysis to evaluate the activities of EGFR and its downstream effectors AKT, ERK, and STAT3 in ESCC; we also analyzed the relationships of these markers and their association with prognosis in ESCC patients.

## Materials and methods

### Study population

We have performed a series of studies to explore the clinical and biological prognostic factors in thoracic ESCC in patients who underwent complete three-field lymphadenectomy (3FLND) [11, 12]. We reviewed the pathology reports of all patients with EC who underwent 3FLND at our hospital between 2001 and 2009, and 354 patients were selected on the basis of the following clinical criteria: having pathologically confirmed thoracic ESCC; having only one primary tumor; not receiving preoperative chemotherapy and/or radiotherapy; having undergone 3FLND with ≥15 total lymph nodes removed; and having tumor-free resection of margins by microscopic examination of the surgical specimen. Of these patients, 22 were excluded from analysis because of perioperative deaths (2 patients) and lost to follow-up (20 patients). Among the remaining 332 patients, paraffin specimens were not available for 57; thus, 275 patients were selected for this study.

The preoperative workup, surgical procedure, and criteria for adjuvant treatment and follow-up were described elsewhere [11, 12]. The clinicopathologic characteristics of the study population are summarized in Table 1. This study was approved by the Institutional Review Board, which waived the requirement for written informed consent of individual patients, given the retrospective nature of this study.

**Table 1:** Clinicopathologic characteristics of 275 patients included in our study

Characteristics	No. of patients (%)
Sex	
Male	223 (81.1)
Female	52 (18.9)
Age (year) (36–78 year; median 57 year)	
≤60	180 (65.5)
<60	95 (34.5)
Tumor location	
Upper	37 (13.5)
Middle	175 (63.6)
Lower	63 (22.9)
Tumor length	
<5 cm	123 (44.7)
≥5 cm	152 (55.3)
Tumor differentiation	
Well differentiated	26 (9.5)
Moderately differentiated	184 (66.9)
Poorly differentiated	65 (23.6)
Pathologic T stage	
T1	13 (4.7)
T2	100 (36.4)
T3	127 (46.2)
T4	35 (12.7)
Pathologic N stage	
N0	101 (36.7)
N1	74 (26.9)
N2	71 (25.8)
N3	29 (10.5)
Pathologic TNM stage	
I	11 (4.0)
II	120 (43.6)
III	144 (52.4)
Adjuvant therapy	
None	106 (38.5)
Radiotherapy	10 (3.6)
Chemotherapy	83 (30.2)
Chemoradiotherapy	50 (18.2)
Unknown	26 (9.5)

### TMA construction and IHC analysis

**Fig. 1:**
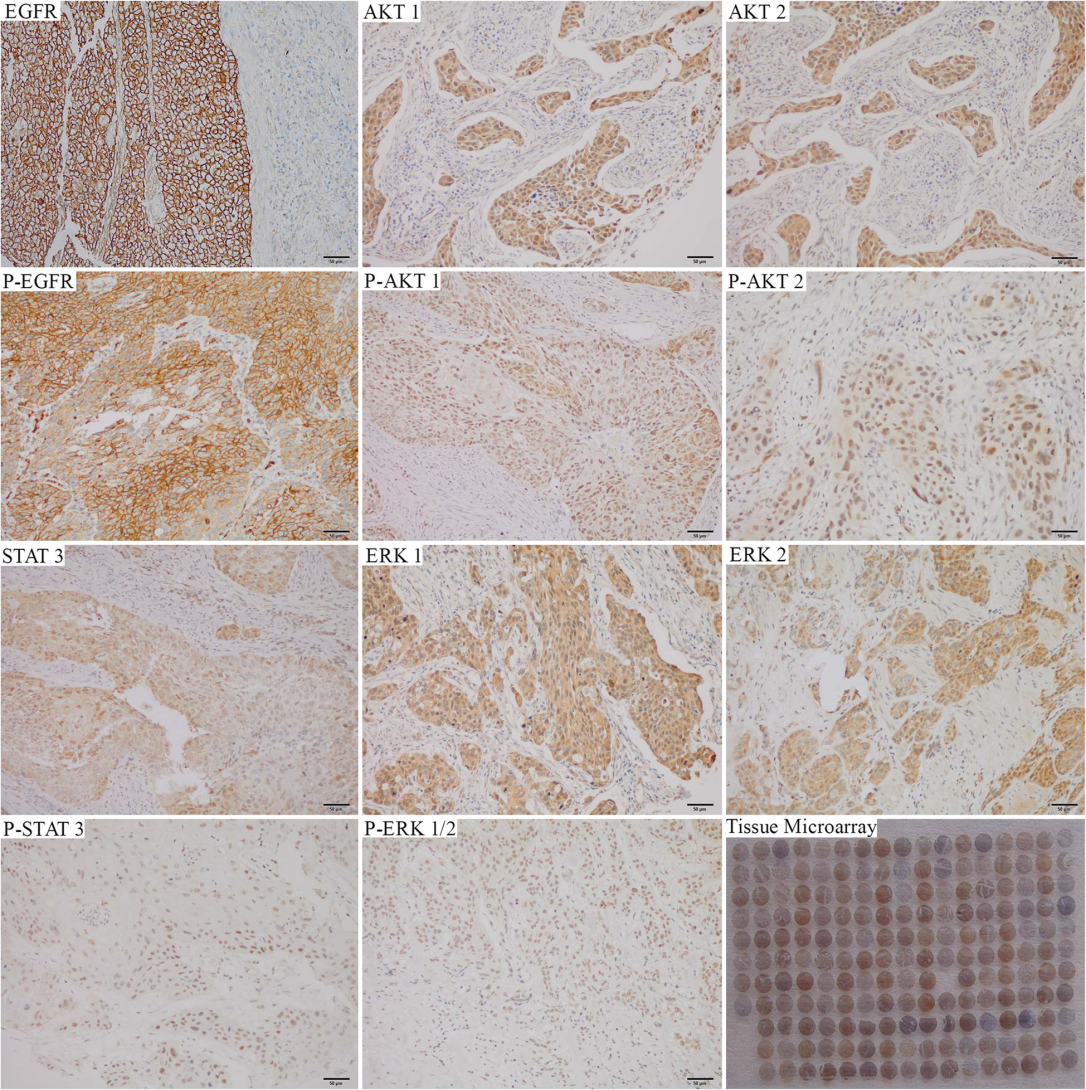
Representative findings on immunohistochemical staining for the tested biomarkers (original magnification × 200): EGFR, phosphorylated (p)-EGFR, AKT1, p-AKT1, AKT2, p-AKT2, ERK1, ERK2, p-ERK1/2, STAT3, and p-STAT3

TMAs were constructed in collaboration with the Department of Pathology at our hospital according to established methods [13]. For each patient, the tumor was identified on the original hematoxylin and eosin-stained (H&E) slides, and the corresponding formalin-fixed, paraffin-embedded tissue blocks were obtained. With use of a UATM-272A Tissue Microarrayer (Unitma, Seoul, Korea), three 1-mm tissue cores which were punched from various areas of the predominant tumor population and one 1-mm normal tissue cone which were punched from the normal areas around tumor for each patient and deposited into a 12 ×10 TMA block (120 cores). IHC staining was performed on 4-μm paraffin-embedded sections from TMA blocks by the standard Envision method using a panel of antibodies: EGFR (113, dilution 1:50; Dako), AKT1 (C73H10, dilution 3 μg/ml; Cell Signaling), AKT2 (302501, dilution 25 μg/ml; R&D), ERK1 (Y72, dilution 1:100; Abcam), ERK2 (E460, dilution 1:250; Abcam), STAT3 (E121-21, dilution 1:50; Abcam), phosphorylated-EGFR (p-EGFR) (Tyr1068) (EP774Y, dilution 1:250; Abcam), phosphorylated-AKT1 (p-AKT1) (Ser473) (EP2109Y, dilution 1:100; Abcam), phosphorylated-AKT2 (p-AKT2) (Ser474) (D3H2, dilution 1:100; Cell Signaling), phosphorylated-ERK1/2 (p-ERK1/2) (MAPK-YT, dilution 1:100; Abcam), and phosphorylated- STAT3 (p-STAT3) (EP2147Y, dilution 1:250; Abcam) (Fig. 1).

### IHC scoring

A modified semiquantitative method H-score was used to evaluate IHC staining [14, 15]. For each tissue core, a score was generated by multiplying the percentages of positive cells (0–100 %) and the intensity of staining. For EGFR and p-EGFR, the staining intensity was classified as follows: 0, no staining; 1+, partial membrane staining; 2+, weak, complete membrane staining; 3+, moderate, complete membrane staining; and 4+, strong, complete membrane staining. For the other markers, the staining intensity for IHC reaction was classified as follows: 0, negative; 1+, weak; 2+, moderate; and 3+, strong. Thus, the overall H-score ranged from 0 to 400 (EGFR or p-EGFR) or 0 to 300 (the other markers). All immunostains were evaluated independently by three pathologists and discordant cases were reevaluated; consensus was reached with use of a multiheaded microscope.

### Statistical analysis

Continuous variables were summarized by descriptive statistics, such as means, standard deviations (SD), medians, and ranges. Categorical variables were tabulated by frequency and percentage. The survival functions were computed from the date of surgery by using Kaplan-Meier estimates, and the log-rank test was used to assess the equality of survival functions. Spearman rank correlation tests were used to assess the relationships among protein expression. Since there’s at present no consensus which cut-off points were best for the markers we tested, we arbitrarily chose the median H-score values as the cut-points for the categorical analyses: the marker was considered high expression with the H-score of ≥ the median value, and low expression with the H-score of < the median value. The univariate and multivariate Cox regression analyses were performed to test for the independent influence of potential prognostic factors on overall survival (OS). Probability (P) values <0.05 were considered statistically significant, and statistical tests were based on a two-sided significance level. Statistical analyses were performed with use of Statistical Package for the Social Sciences software (SPSS, Chicago, IL).

## Results

### Correlation between EGFR expression and AKT, ERK and STAT3 in ESCC

EGFR and p-EGFR staining were predominantly located in the cell membrane. AKT1, AKT2, ERK1, ERK2, and STAT3 immunoreactivity was mainly located in the cytoplasm. p-AKT1, p-AKT2, p-ERK1/2, and p-STAT3 expression was detected in both the cytoplasm and nucleus. Due to the inevitable loss of biopsy cores or insufficient tumor cells present in the cores, about 2–5 cases were missed for each marker staining. The patients who missed any data of marker staining were excluded, and left 270 patients for the final analysis. All of the marker expression results are summarized in Table 2.

**Table 2:** Expression of proteins in quartiles of H-scores

	Median	Minimum	Maximum	25 %	75 %
EGFR	80	0	400	0	160
p-EGFR	10	0	400	0	50
AKT1	30	0	240	5	80
p-AKT1 (Ser473)	70	0	250	40	100
AKT2	0	0	120	0	20
p-AKT2 (Ser474)	0	0	80	0	10
ERK1	40	0	210	0	60
ERK2	70	0	250	30	100
p-ERK1/2	50	0	170	30	80
STAT3	50	0	300	30	80
p-STAT3	30	0	300	10	60

Results from Spearman rank correlation analyses among EGFR, p-EGFR, p-AKT1, pAKT2, p-ERK1/2, and p-STAT3 showed that EGFR expression was correlated with that of p-EGFR (*P* = 0.001), p-AKT1 (*P* < 0.001) and p-AKT2 (*P* < 0.001) but not with that of p-ERK1/2 (*P* = 0.630) or p-STAT3 (*P* = 0.835); p-EGFR expression was correlated statistically with that of p-AKT1 (*P* < 0.001), p-AKT2 (*P* < 0.001), p-ERK1/2 (*P* = 0.027), and p-STAT3 (*P* < 0.001). p-AKT1, p-AKT2, p-ERK1/2, and p-STAT3 expressions was correlated with each other except for that between p-AKT2 and p-ERK1/2 (Table 3).

**Table 3:** Correlations of the protein expression: Spearman rank correlation tests

		EGFR	p-EGFR	p-AKT1	p-AKT2	p-ERK1/2	p-STAT3
EGFR	Correlation coefficient	1.000	0.196	0.269	0.226	−0.030	0.013
	*P* value	.	0.001	<0.001	<0.001	0.630	0.835
p-EGFR	Correlation coefficient	0.196	1.000	0.337	0.331	0.135	0.390
	*P* value	0.001	.	<0.001	<0.001	0.027	<0.001
p-AKT1	Correlation coefficient	0.269	0.337	1.000	0.355	0.127	0.219
	*P* value	<0.001	<0.001	.	<0.001	0.038	<0.001
p-AKT2	Correlation coefficient	0.226	0.331	0.355	1.000	0.018	0.180
	*P* value	<0.001	<0.001	<0.001	.	0.772	0.003
p-ERK1/2	Correlation coefficient	−0.030	0.135	0.127	0.018	1.000	0.204
	*P* value	0.630	0.027	0.038	0.772	.	0.001
p-STAT3	Correlation coefficient	0.013	0.390	0.219	0.180	0.204	1.000
	*P* value	0.835	<0.001	<0.001	0.003	0.001	.

### Clinical significance of phosphorylated-AKT1 in ESCC

**Fig. 2:**
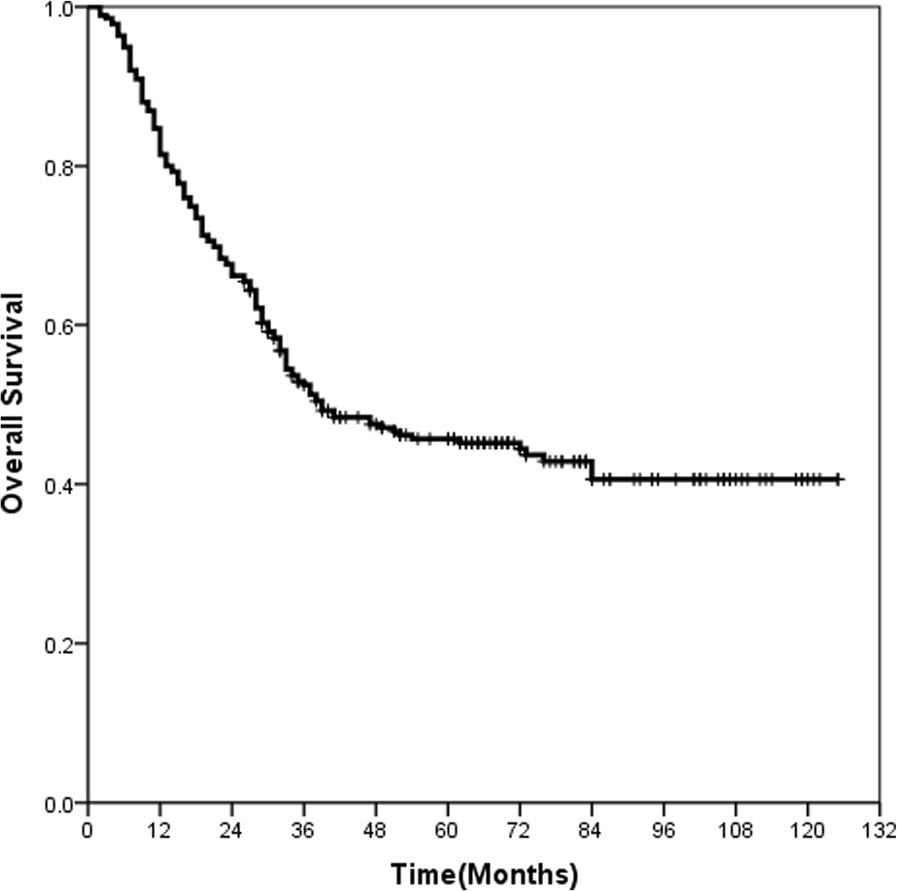
Kaplan-Meier curve of overall survival for all patients

**Fig. 3:**
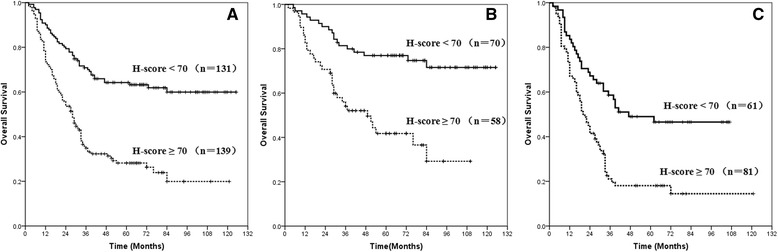
Log-rank tests of overall survival comparing patients with p-AKT1 H-scores of ≥70 and those with p-AKT1 H-scores of <70 for (**a**) all patients (*n* = 270; *P* < 0.001); **b** stage I-II patients (*n* = 128; *P* < 0.001); and **c** stage III patients (*n* = 142; *P* < 0.001)

At a median follow-up time of 34 months (range: 2–125 months), the median OS for the entire cohort was 39 months (95 % confidence interval [CI]: 20–58 months), and survival rates were 52.5 % at 3 years and 45.2 % at 5 years (Fig. 2). The variables tested on univariate analysis showed that the factors that were significantly associated with OS included gender, N stage, adjuvant therapy, and expression of p-AKT1 (Table 4). On multivariate analysis, gender, N stage, and p-AKT1 expression were found to be the independent prognostic factors for OS (Table 4). When expression of p-AKT1 increased, patients’ survival duration decreased (HR: 2.682, 95 % CI: 1.891–3.802). Log-rank tests of overall survival comparing patients with p-AKT1 high expression (H-scores ≥ 70) and those with p-AKT1 low expression (H-scores < 70) show that the group with p-AKT1 low expression had significantly better OS than did the group with high expression among all patients (*P* < 0.001, Fig. 3a), patients with stage I-II diease (*P* < 0.001, Fig. 3b) and patients with stage III disease (*P* < 0.001, Fig. 3c).

**Table 4:** Univariate and multivariate for overall survival: Cox proportional hazards regression model

Prognostic factors	OS		
	*P* value	*P* value	HR (95 % CI)
	(univariate)	(multivariate)	(for multivariate)
Sex (male, female)	0.011	0.012	1.346 (1.071-1.803)
Age (≤60, >60 year)	0.373		
Tumor location (upper, middle, lower)	0.277		
Tumor length (<5 cm, ≥5 cm)	0.890		
Tumor differentiation (well, moderately, poorly)	0.148		
T stage (T1, T2, T3, T4)	0.128		
N stage (N0, N1, N2, N3)	<0.001	<0.001	
N0			1
N1			1.875 (1.187–2.962)
N2			3.646 (2.355–5.645)
N3			2.444 (1.387–4.307)
Adjuvant therapy (none, chemotherapy, radiotherapy, chemoradiotherapy, unknown)	0.027		
EGFR (≥80, <80)	0.735		
p-EGFR (≥10, <10)	0.392		
AKT1 (≥30, <30)	0.362		
p-AKT1 (≥70, <70)	<0.001	<0.001	2.682 (1.891–3.802)
AKT2 (>0, =0)	0.179		
p-AKT2 (>0, =0)	0.379		
ERK1 (≥40, <40)	0.683		
ERK2 (≥70, <70)	0.558		
p-ERK1/2 (≥50, <50)	0.209		
STAT3 (≥50, <50)	0.233		
p-STAT3 (≥30, <30)	0.621		

HR, hazard ratio; CI, confidence interval; OS = overall survival

## Discussion

In this study, we tested the protein expression and activities of EGFR as well as several key nodes on its downstream pathways for ESCC patients and found that expression of p-AKT1, p-AKT2, p-ERK1/2, and p-STAT3 was significantly related to the expression of p-EGFR. We also found that activation of AKT1 independently influenced patients’ survival, with higher expression of p-AKT1 being linked to poorer OS; neither EGFR nor p-EGFR expression, however, had a prognostic effect in ESCC patients in this cohort.

Currently, the role of EGFR in tumor development in ESCC is not clear, although elevated expression of EGFR has been reported in 50–90 % of patients with ESCC [16–20]. Several studies have shown that EGFR overexpression was associated with poor OS and poor disease-free survival in ESCC patients [6–8]; other studies, however, did not find a prognostic effect for EGFR overexpression in ESCC [9, 10]. The inconsistent conclusions drawn from the various studies might be due to differences in patient selection, treatments, and the methods used for detecting and scoring EGFR expression.

In the present study, the prognostic value of EGFR was not found. Several researchers [9, 21] have reported that EGFR expression has predictive value for the therapeutic effect of chemotherapy and radiotherapy in ESCC; specifically, patients with elevated EGFR expression had better treatment outcomes after chemoradiotherapy. To rule out the influence of adjuvant chemotherapy and/or radiotherapy in the prognosis, we performed Cox regression analyses for the 106 patients who did not receive adjuvant therapy in our group and still found no association between EGFR expression and OS (data not shown). Our future work will explore whether alterations of EGFR, including gene mutation and amplification, have prognostic values in ESCC.

EGFR is a tyrosine kinase receptor, and the phosphorylated tyrosine residue serves as a docking site to exert its biological roles. After being phosphorylated, EGFR is activated and then in turn activates multiple downstream intracellular signaling pathways, mainly PI3K-Akt, STAT3, and Ras-Raf-MAPK pathways. Our finding that p-EGFR was highly correlated to the phosphorylation of AKT1, AKT2, ERK1/2, and STAT3 indicated that p-EGFR possibly contributed to the activation of these downstream pathways in ESCC, suggesting that the EGFR pathways might be active in some patients with ESCC. However, we also observed that p-AKT1 could predict the prognosis of ESCC, while EGFR and p-EGFR could not be, suggesting that the activation of AKT1 resulted from other factors in some patients. Besides EGFR stimulation, several other ways of activating AKT1 have been reported, including other growth factor receptors such as VEGF and PDGF, mutations of PI3K or RAS, inactivation of tumor suppressor gene PTEN, and AKT1^E17K^ somatic mutations [22–26]. The exact mechanisms of this phenomenon in ESCC are unclear and need further investigation.

AKT, a serine/threonine protein kinase, is the central mediator of the canonical PI3K pathway, which can mediate various cellular functions including cell metabolism, growth, proliferation, survival, apoptosis, and angiogenesis [27]. A number of studies have demonstrated the overactivation of AKT in many human solid tumors and hematological malignancies [28]. AKT has three isoforms: AKT1, AKT2, and AKT3. Although these AKT family members share a similar domain structure, they have distinct substrates and different physiological behaviors [29]. These AKT isoforms seem to mediate different functions in cancer pathophysiology; for example, AKT1 appears to promote mammary tumor induction, whereas AKT2 promotes metastasis in previous reports [29, 30]. This may explain the difference in the prognostic effects between p-AKT1 and p-AKT2 in ESCC patients in our study.

The prognostic values of p-AKT1 have been studied for several malignancies. Interestingly, many studies have shown that activation of AKT1 was associated with poor prognosis [31–33], whereas other studies have shown AKT1 activation to be a favorable prognostic indicator [34–36]. To the best of our knowledge, few studies have examined the association between AKT1 activation and clinical outcome in ESCC. Yoshioka et al. [37] used IHC analysis to examine p-AKT expression in 235 ESCC patients who underwent surgery with or without preoperative chemotherapy and found that p-AKT expression was associated with poor prognosis in those who had received chemotherapy but did not correlate with survival in those who had not received chemotherapy. However, that study did not specify the isoform of AKT1. Nowadays, the PI3K/AKT pathway has been recognized as an important pathway in the development of cancers [38]. Our study suggested the potential of AKT1 as a target for anticancer therapeutics in ESCC.

## Conclusion

Our study suggests p-AKT1 is associated with poor prognosis in patients with ESCC, and supports further studies to investigate the potential mechanisms.

## Abbreviations

EC: Esophageal cancer
ESCC: Esophageal squamous cell carcinoma
EGFR: Epidermal growth factor receptor
TMA: Tissue microarray
IHC: Immunohistochemical
3FLND: Three-field lymphadenectomy
H&E: Hematoxylin and eosin-stained
p-EGFR: Phosphorylated-EGFR
p-AKT1: Phosphorylated-AKT1
p-AKT2: Phosphorylated-AKT2
p-ERK1/2: Phosphorylated-ERK1/2
p-STAT3: Phosphorylated- STAT3
SD: Standard deviations
OS: Overall survival
CI: Confidence interval
